# Instructional design communication through AI-assisted storytelling: a hybrid model grounded in educational psychology

**DOI:** 10.3389/fpsyg.2026.1785568

**Published:** 2026-09-03

**Authors:** Yifan Pan, Muhammad Zammad Aslam

**Affiliations:** 1 Shanghai Publishing and Printing College, Shanghai, China; 2 School of Languages, Literacies and Translation, Universiti Sains Malaysia, George Town, Penang, Malaysia

**Keywords:** artificial intelligence (AI) in instructional design, collaboration tools, digital instructional design communication, educational communication, stakeholder engagement, storytelling in instructional design, virtual and augmented reality (VR/AR)

## Abstract

**Introduction:**

Artificial intelligence (AI)-assisted storytelling is increasingly transforming educational communication and instructional design, particularly in isolated and remote learning environments. However, challenges related to collaboration, meaningful interaction, technological accessibility, and ethical use remain. Drawing on educational psychology, this study investigates how AI-assisted storytelling communication strategies can support cognitive engagement, motivation, creativity, collaboration, and stakeholder engagement in AI-supported educational communication systems. It also examines how digital communication technologies are restructuring instructional design communication and how storytelling can be integrated with AI-mediated communication to address emerging educational challenges.

**Methods:**

This qualitative study employed narrative interviews with 16 digital instructional designers and AI developers selected through purposive sampling. Data were collected through semi-structured interviews, case studies, and content analysis of relevant instructional design and educational communication practices. The collected data were analyzed using thematic analysis to identify recurring patterns related to instructional design communication, educational communication, storytelling practices, digital collaboration, and AI-mediated communication.

**Results:**

The findings indicate that although digital technologies have facilitated communication and collaboration among educational teams, particularly in remote environments, they may also reduce opportunities for deep interpersonal interaction. Technologies such as Artificial Intelligence (AI), WeChat, and VooV Meeting have enhanced communication dynamics but have introduced challenges, including the absence of non-verbal cues and unequal access to technology. Emerging technologies such as AI, Virtual Reality (VR), and Augmented Reality (AR) are reshaping instructional design by creating more engaging learning experiences; however, concerns regarding ethical use, accessibility, equity, and fairness remain. Storytelling emerged as an effective strategy for strengthening educators' engagement, improving understanding, fostering creativity, and promoting stakeholder collaboration.

**Discussion/Conclusion:**

The findings suggest that AI-assisted storytelling can provide a meaningful communication strategy for addressing the collaborative and interactional limitations of digitally mediated educational environments. The study therefore proposes the "Hybrid Storytelling-Digital Communication (HSDC) Model", which integrates storytelling with AI-supported digital communication tools to promote meaningful interaction, creativity, collaboration, and stakeholder engagement while addressing ethical, accessibility, and equity concerns.

## 1 Introduction

Communication has long been a keystone in the process of instructional design, serving as a bridge to share, refine, and ultimately apprehend ideas (Branch and Clark-Stallkamp, 2023; Brown and Green, 2019; Børte and Lillejord, 2024). By tradition, this communication occurred through direct interactions, plans, and physical models, which enabled instructional designers and stakeholders to team up directly (Abdullah et al., 2024; Cross, 1982; Jones, 2024; Schön, 2017). The methods rely greatly on implicit knowledge and personified interaction, encouraging profound collaboration and instant feedback processes (Abdullah et al., 2024). This collaborative interaction reflects social-constructivist principles (Vygotsky, 1978; Bruner, 1990; Kim et al., 2022), as learners and designers create meaning together through facilitated dialogue (Ataş A and Yildirim, 2025; Cober et al., 2015; Nielsen et al., 2003). However, the emergence of digital technologies has not disrupted this setting but instead enhanced it, introducing and bringing new and novel tools that boost flexibility, speed, and the geographic grasp of instructional design collaboration.

Whereas digital technologies all over the world have emerged as strong tools of communication (Iqbal et al., 2025; Nguyen et al., 2025; Dwivedi et al., 2021), they have transfigured instructional design alliances as well (Nørgård, 2021; St John and Suhendra, 2024). Due to their powerful role, their impact on stakeholders' engagement in faraway surroundings, ingenious imagination, and unconstrained problem-solving remains unexplored to a large extent (Kenny and Castilla-Rho, 2022; Ye et al., 2022). Moreover, prior studies frequently focus attention on the technical capabilities of these tools in lieu of their emotional and cognitive impressions (Duin and Pedersen, 2023; Hartner-Tiefenthaler et al., 2022). Besides, the strategic significance of narrative has not been satisfactorily acknowledged in theoretical models of design communication. The aim of this study is to address knowledge gaps by scrutinizing the influencing impact of digital communication technologies, narratives, and AI on instructional design involvement.

Contemporary research-based studies emphasize the role played by digital tools, such as WeChat, VooV Meeting, and a few other platforms, in the restructuring of instructional design and educational communication. Jia et al. (2023) argue that the appearance and availability of the platforms have made isolated collaboration innovative and modern. This has been made possible by permitting instructional design teams to work across distances while maintaining actual communication. However, from the perspective of educational psychology, greater dependence on fast digital communication may increase unnecessary cognitive demands (Pohn et al., 2025; Sweller, 1994), which could potentially constrain deeper learning and knowledge integration (Biju et al., 2024; Mayer, 2005). Likewise, Duin and Pedersen (2023) emphasize the implications of AI while analyzing users' responses; nevertheless, they do not concede the technology's margins in procuring implicit instructional design understanding, in spite of the fact that it is a decisive element of the creative procedure (Cross, 1982, 2006). This conflict between swift data analysis and profound conceptual insights highlights a significant challenge in digital learning settings (Li and Zhang, 2025).

The natural and iterative nature of instructional design and educational communication is disregarded owing to the reliance on AI on quantitative metrics. These kinds of technological progressions have endorsed further captivating communication approaches and methods, together with the usage of virtual reality (VR) and augmented reality (AR), to envisage multifaceted instructional design perceptions. The potential of these technologies to revolutionize design communication is inspiring (Bibri and Jagatheesaperumal, 2023; Rajaratnam et al., 2022; Reiners et al., 2021).

Nevertheless, despite these mentioned advances and developments, there exist many challenges that need to be addressed. A mutual concern indicates that digital tools have the ability to control non-verbal signs, for instance, facial expressions and body language, considered vital in understanding the (instructional) design response fully (Fares and Jammal, 2023). Furthermore, doubtlessly, AI, VR, and AR deliver notable potential in improving and strengthening communication; they are inclined to generate challenges regarding accessibility as well as the necessity for user training (Hartner-Tiefenthaler et al., 2022; Lorié et al., 2017; Levand, 2020). Inequities in access impact inclusion and continue to sustain achievement gaps, from an educational psychology viewpoint (Kulal et al., 2024). These discussed specific issues point out the demand for further research in this field, declaring how the said tools intend to affect the overall instructional design process, specifically in terms of association, creativity, and stakeholder engagement or commitment.

This study primarily aims to explore how communication evolves to support realistic instructional design experiences by integrating digital technology with storytelling. The study has specific aims/objectives: first, to analyse how digital communication technologies reshape the instructional design process; second, to assess how storytelling can improve stakeholder engagement and comprehension. In this respect, the present research will address two key questions: 1. How can storytelling techniques be successfully incorporated with AI-guided digital instructional design and educational communication? 2. What effects does the HSDC model have on communication clarity and creative collaboration? Additionally, the study will examine the advantages and challenges associated with using new technology in instructional design, communication, and educational communication, and how these communication strategies influence the creative process, teamwork, and the successful execution of instructional design projects.

The present study is informed by several complementary theoretical perspectives. Social constructivist principles suggest that learners and designers create meaning together through facilitated dialogue and collaborative interaction (Vygotsky, 1978; Bruner, 1990; Kim et al., 2022). Cognitive Load Theory explains how communication strategies and digital technologies may influence learners' processing of information and the development of deeper schemas (Sweller, 1994; Mayer, 2005; Pohn et al., 2025). The study also considers concepts related to self-regulation, motivation, and learner engagement in digital learning environments (Zimmerman, 2002; Bandura, 2002; Deci and Ryan, 2000; Gopez and Gopez, 2024). Furthermore, social presence and collaborative interaction remain important in remote instructional design settings where communication increasingly occurs through digital platforms (Garrison, 2007; Zhang and Zhang, 2024). Together, these theoretical perspectives provide the foundation for examining the role of AI-assisted storytelling and digital communication technologies in instructional design communication and educational communication.

Hartner-Tiefenthaler et al. (2022) are of the view that earlier conducted research has focused on these mentioned technical aspects of tools, ignoring their wider consequences/impacts on user involvement, creativity, and collaboration. The current study merges technological effectiveness and capability with human-centered instructional design and educational communication by amalgamating insights from digital instructional design practices and storytelling design and techniques (Li et al., 2024; Parrish, 2006) to construct an absolute framework for amplifying distant instructional design collaboration so that it is positively responsive.

## 2 Methodology

### 2.1 Research design

This research employs thematic analysis to explore communication, with a focus on instructional design and educational communication. Although the sample size is small, qualitative research emphasizes thematic saturation over statistical generalizability (Guest et al., 2006; Hennink and Kaiser, 2022). This qualitative study utilizes narrative interviews with 16 digital instructional designers and AI developers, selected through purposive sampling. Data were coded based on grounded theory principles, and the approach adhered to Braun and Clarke's guidance that interviewing this number of participants can yield meaningful insights.

Researchers aimed to gather diverse perspectives on the development of the Pinterest, Tieba, and REDnote websites. Based on the principles of educational psychology, the interview questions concentrated on investigating how participants perceived learning outcomes along with their cognitive engagement while completing design activities. Comparable qualitative methods have been successfully utilized in previous research to investigate digital learning practices (Hennink and Kaiser, 2022).

To enhance reliability, an investigator triangulation approach was employed, integrating case studies and findings from online content analysis. Additionally, to address potential researcher bias, reflexivity journals and member validation techniques were applied to verify participant responses.

Themes based on Iterative analysis enable a comprehensive examination of how communication tends to influence the instructional design process. Due to its adaptability and flexibility, this approach is appropriate for assessing instructional design, communication, and educational communication's complicated and diverse character. The present study focuses on topics to identify patterns and correlations in the data. The observed relationships and patterns can offer valuable insights into the role of communication in the instructional design field.

### 2.2 Participants and study context

In this regard, to collect data, interviews were conducted with 16 diverse participants, including instructional designers from many educational fields, such as graphic-based instructional design, learning content selection instructional design, learner experiences, and user interface instructional design, as well as learners/students and shareholders from several commercial sectors that existed in Shanghai, China.

In the study, participants with different professional backgrounds were selected so that the study could reflect varied perspectives on communication in instructional design. AI-mediated speech identification and real-time captioning further improve the reliability of interaction by minimizing misunderstandings in the domain of virtual collaboration (Akbar et al., 2022; Mariappan and Krishnan, 2023).

The current study conducted semi-structured interviews with instructional designers (formerly known as curriculum designers), educators, and learners/students involved in diverse instructional settings.

Written informed consent was obtained from all the participants. After the completion of the process of informed consent, the Institutional Review Board of SPP College granted approval for the present study.

### 2.3 Data collection

The present study collected data through semi-structured interviews conducted between 20 October 2024 and 18 December 2024, detailed case studies, and careful comprehensive content analysis.

**Table 1:** Participant profile and sampling information.

Participant category	Final participants (*n*)	Age range (years)	Professional experience
AI Developers	4	28–33	More than 3 years
Instructional Designers	4	28–38	More than 3 years
Educators	3	29–54	3–15 years
Learners/students	3	24–28	Not Applicable
Stakeholders	2	29–45	3–10 years
**Total**	**16**	—	—

A total of 25 individuals were initially approached through purposive sampling. Data collection continued until thematic saturation was achieved after 16 interviews. Consequently, the final analysis included 16 participants representing AI developers, instructional designers, educators, learners/students, and stakeholders from commercial sectors in Shanghai, China (see Table 1 for details).

The semi-structured interviews included focused questions in order to explore participants' insights related to emotional responses of the learners, their self-efficacy beliefs, and changes in motivation during the usage of digital technologies/tools. Such a strategy remains consistent with qualitative research in educational psychology, indicating the significance of learners' emotions and self-directed learning (Alhazmi and Kaufmann, 2022). The interview guide for the semi-structured interviews was designed in line with the study objectives and its main theme-centered areas. Accordingly, the full protocol has been provided in Appendix A.

However, the participants' experiences with communication tools and tactics were the main emphasis during the interviews. Kvale (1996) and Alhazmi and Kaufmann (2022) suggested a technique that facilitates detailed consideration of the perspectives and experiences of the participants under analysis. However, it also offers the required adaptability in exploring the evolving topics as they are found or discovered.

### 2.4 Case studies and online content sources

Correspondingly, the section steps ahead with the purpose of proposing an inclusive perception of the implication and execution of communication strategies in selected instructional design settings, followed by practical backgrounds.

This collection of case studies, however, consisted of projects that comprehensively utilized digital communication tools and storytelling techniques and methods.

A selection of case studies was made based on their pertinence to the described research objectives, while overtly showing the focus on the projects that included considerable cooperation, collaboration, and innovation in communication methods or techniques.

Similarly, the collection and analysis of the selected research-related material from online instructional design platforms and forums were obtained to boost the overall conception and perception of effective instructional design communication.

The remarkable instructional design, communication, and educational communication in communities, together with REDnote, Pinterest, and Tieba, existed among the platforms that were used.

These kinds of forums serve as a facilitation for platforms where expert instructional designers frequently exchange their specific work and deliberate challenges and strategies in their communication to make it influential.

The content-based analysis aims to expose repeated problems and grave topics connected to instructional design, communication, and educational communication.

Additionally, other examples of similar subjects and themes include the application and usage of digital technologies, the prominence of narrative, and the impression of rising technology.

### 2.5 Data analysis process

In this section, the researcher did the theme-based analysis following the procedure suggested by Braun and Clarke (2006) and (Bingham, 2023).

Keeping in view the prescribed procedure, the data underwent the coding step to recognize the repeated themes set as part of the current study (see Aslam et al., 2023).

Then, these selected themes were categorized into wide-ranging groups in lieu of different instructional design, communication, and educational communication facets.

The same process involved several stages, which comprised familiarizing oneself with the data, creating initial codes, categorizing themes, evaluating themes, defining and categorizing themes, and validating the study. To enhance methodological transparency, Table 2 presents illustrative examples of the thematic coding process employed in this study. The table demonstrates how representative participant quotations were interpreted into initial codes and subsequently grouped into broader analytical categories following thematic analysis approach (Braun and Clarke, 2006; Bingham, 2023; Sandars et al., 2024). These examples are provided to illustrate the coding procedure and analytical process rather than to present the complete coding framework developed during the thematic analysis.

**Table 2:** Illustration of the thematic coding process.

Representative participant quotation	Illustrative initial code	Analytical category
“*Video conferencing is fast and convenient, but it cannot fully convey facial expressions and other subtle cues that help us understand each other.”* (P1)	Limited non-verbal communication	Digital communication challenges
“*Stories help learners connect the content to real situations, making the learning experience more meaningful and memorable.”* (P5)	Storytelling supports meaningful learning	Narrative engagement
“*Digital tools make collaboration possible across locations, but they also require stronger communication skills to avoid misunderstandings.”* (P6)	Communication required for remote collaboration	Digital collaboration
“*Artificial intelligence has the potential to support decision-making, but having dependence on it too greatly may minimize creativity and human judgment in the design process.”* (P1)	Concern about overreliance on AI	AI integration challenges
“*Visual representations (diagrams, sketches) assist in simplifying complex ideas and making communication more effective for both designers as well as learners.”* (P1)	Visuals improve understanding	Visual communication
“*AI has the capability to personalize communication in an effective way, but users need to be aware of the way their data are collected, safely stored, and utilized in research.”* (P1)	Privacy and transparency concerns	Ethical considerations

The complete coding process involved iterative refinement of codes through repeated engagement with the interview transcripts, constant comparison across participants and supporting data sources, and the development of higher-order analytical categories that informed the seven themes presented in Section 3. This iterative process enhanced the credibility and trustworthiness of the thematic analysis.

Coding focused on emerging categories concerning learners' motivation, self-efficacy, and cognitive load in digital design collaborations.

Accordingly, the analysis investigated each issue in relation to the instructional design process to understand how digital tools and narrative strategies shaped and influenced creativity, collaboration, and stakeholder engagement. Based on the analysis, the findings were then compared with the contemporary literature to identify areas of agreement and divergence.

Somehow, the current study utilized a thematic analysis with a cross-case comparison. This comparison involved an investigation of themes identified in one case study as compared to those recognized in other case studies in order to identify shared tendencies, patterns, and differences (Aslam et al., 2024; Alyaqoub et al., 2024).

The application of the same approach enabled the researchers to identify the factors that enhance the effectiveness of instructional design and educational communication, along with the challenges that need to be focused on.

### 2.6 Reproducibility

In order to enhance reproducibility in the research findings, thematic categories were systematically positioned and then coded in all selected stories.

Such a systematic categorization aimed to include identifying as well as authenticating main themes like AI-assisted empathy, instructional design coherence, and narrative immersion.

An audit trail was maintained in accordance with educational psychology values and included reflective field notes documenting participants' self-regulation as well as their motivational understandings (Bingham, 2023).

The themes identified were well explored and reinforced by two coders, which enhanced the reliability and objectivity of the thematic analysis.

Such a systemic approach adds validity to the results and facilitates an in-depth and fine-grained analysis of the underlying stories.

### 2.7 Pilot application of the HSDC model

**Figure 1:**
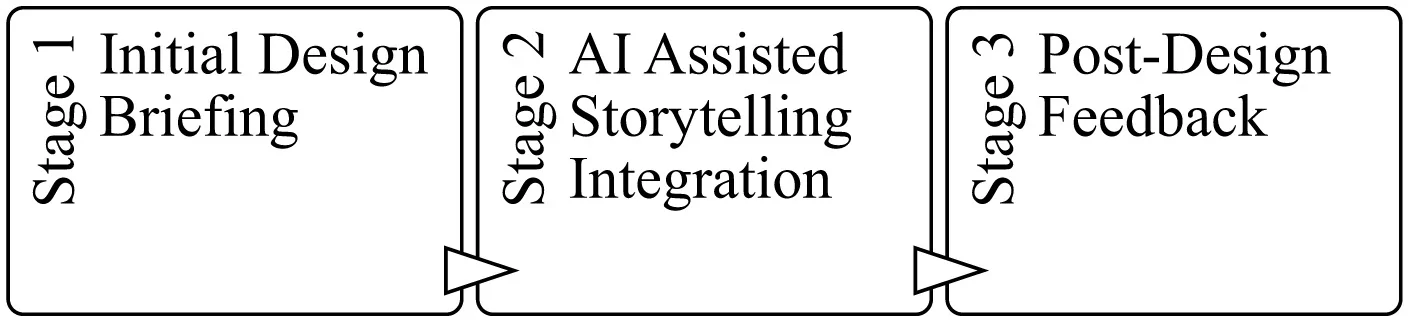
Pilot application of HSDC model. Source: Author-developed.

To demonstrate the practical feasibility of the proposed HSDC model, an exploratory pilot application was conducted with two teams of digital instructional designers (*n* = 6). The pilot was intended to illustrate the model's initial implementation rather than to provide formal empirical validation. The pilot was not intended to provide statistical validation of the model but rather to explore its initial application prior to larger-scale empirical investigation (Sandars et al., 2024; Trevisan et al., 2024; Oates and Mynott, 2025). Learner outcomes, including task persistence, self-efficacy, and reflective journal entries, were collected to evaluate the educational impact (Rohles et al., 2022). The process was divided into three phases: (1) early instructional design briefing, (2) story AI integration, and (3) feedback after instructional design (see Figure 1) (see Covello, 2025).

In Stage 1, participants were asked to express their instructional design intention in common technical terms. In Phase 2, they leveraged a storytelling scaffold provided by a generative AI tool (ChatGPT/DeepSeek) to reframe their instructional design with a humanistic narrative. In Step 3, feedback was generated through reflexive interviews and analyzed using thematic coding.

## 3 Results

This chapter comprises the process of thematic analysis through which many remarkable themes connected with instructional design, communication, and educational communication were identified. These subjects show insight and understanding into the impact and impression of digital tools and storytelling on the arranged instructional design process concerning engagement, inventiveness, and teamwork among the educators who are inclined to bring changes in communication.

### 3.1 Impact of digital tools on instructional designer-learner communication

It has already been discussed that digital tools have revolutionized cooperation in graphic instructional design and other digital works. Similarly, the advancement of digital communication channels has remarkably amplified the efficacy and pace of interactions between instructional designers, learners/students, and stakeholders. Even so, the analysis revealed that these indicated protocols might sometimes lead to misapprehensions, as there are no existing non-verbal cues or signs. These gaps reduced learners' sense of connection and engagement in the instructional design process.

For instance, P1 reported that while video teaching/conferencing tends to enable quick responses, it often lacks the subtleties that exist in in-person classes, thereby enhancing the chance of misunderstandings. As P1 noted,

“*Video conferencing is fast and convenient, but it cannot fully convey facial expressions and other subtle cues that help us understand each other.”*

Furthermore, the ease with which prompt messaging services enable users to convey short-lived messages was emphasized as having both advantages and disadvantages. P2 and P4 indicated that communication is often fragmented and lacking in depth. As P2 explained,

“*Messages are exchanged quickly, but sometimes the deeper meaning behind the discussion is lost.”*

P3, who belonged to the education field, reported that discussing matters through digital tools does not fully satisfy him because he remains unable to completely understand the resolution. He further explained that, due to limited technical familiarity with digital media technology, he experiences difficulties in perceiving minute details. He also reported challenges related to network unavailability, as he belongs to a remote area where digital signals do not function reliably, causing him to miss the depth of personal communication. According to P3,

“*Poor connectivity and limited technical knowledge often make it difficult to fully understand what is being communicated.”*

Moreover, digital technologies have greatly focused on the concerns related to communication quality. Despite the fact that these technologies facilitate the quick streaming of information, digital tools may not be able to promote the deep and meditative interactions constantly required for multidimensional and complex instructional design projects. Accordingly, P3 and P5 observed that in the absence of physical presence, it could hinder the establishment of relationships, understanding, as well as trust with the learners, as it is vital for successful cooperation. According to the observation of P5,

“*Developing trust and mutual understanding becomes easier when people have an opportunity for physical interaction.”*

Although these digital platforms are advantageous, the findings highlight that instructional designers must show intentional practice in their strategies of communication to ensure the presence of full consistency, cooperation, and engagement among all participants.

### 3.2 Role of storytelling in instructional design engagement

The storytelling strategy has emerged as a prominent approach to increasing the capacity and participation level among the educators. The analysis reveals that educators develop active engagement and a deeper comprehension of the instructional design when the instructional designers apply a storytelling strategy to convey their concepts. One case study illustrated the approach through which an instructional designer used a narrative-based presentation to clarify the significance of newly learned content introduced to learners. As a result, it proved to be a more successful and collaborative approach (Case Study 1, 2023).

Somehow, the effectiveness of the storytelling strategy depends heavily on the capability of the instructional designer in order to attain a satisfying balance between the narrative and visual elements, especially when digital platforms are used. As P5 observed,

“*Stories help learners connect the content to real situations, making the learning experience more meaningful and memorable.”*

Furthermore, storytelling plays a vital role in building emotional relationships between educators and learners. Throughout the engagement in consultation, numerous participants pointed out the significance of a well-sharpened narrative in increasing the memorability and accessibility of an instructional design, thus leading to enhanced support from the connected educators and final users (i.e., learners/students). According to P5,

“*A strong narrative makes instructional content easier to understand and encourages learners to stay engaged throughout the process.”*

Contrariwise, the analysis disclosed the challenges of integrating storytelling in modern digital communication. Using a one-on-one presentation as a demonstration, an instructional designer judged that, even though it is so easy to transmit a story in person in an effective way, it might become more challenging to perform so successfully by means of written communication or virtual classes. In this scenario, P1 reported,

“*Although storytelling is naturally effective in face-to-face communications, sustaining the same level of emotional engagement in virtual environments remains much more difficult.”*

Similarly, according to P5,

“*Since audience involvement cannot be assumed in the same way as in physical environments, digital platforms need various storytelling approaches*”.

Findings of the analysis emphasize that modifying narrative techniques should be used across diverse platforms of communication to ensure that the conveyed message is positively responded to by the audience.

### 3.3 Collaboration and creativity in the digital age

The rise of digital tools has initially changed the model of collaboration in instructional design, enabling teams to cooperate regardless of their geographic location or time zone. However, this growing independence constitutes challenges in upholding coherence and innovation among a detached team, offering one of the correlated problems. Some participants expressed that when digital tools enable agile and iterative instructional design processes, they, too, need extraordinary communication skills to hold the situations or challenges of distant collaboration effectively. As P6 noted,

“*Digital tools make collaboration possible across locations, but they also require stronger communication skills to avoid misunderstandings.”*

Similarly, P13, P12, and P1 emphasized that remote collaboration demands greater coordination and continuous interaction among team members.

Additionally, the study disclosed the necessity of creating flawless communication protocols and clear expectations to guarantee that all team members maintain consensus throughout the given project.

The thematic analysis revealed that digital communication technology could make it easy or obstruct innovative thought. Conversely, these tools enable instructional designers to refine ideas while getting input speedily, providing a way to advance more creative solutions. On the other hand, the desire for physical existence and the potential for broken and isolated communication can both work as hindrances that obstruct the occurrence of innovative collaboration. Many participants observed that brainstorming sessions managed through video conferencing are mostly less practical compared to those conducted physically. This occurrence is generated from the challenge of supporting a constant degree of spontaneity and energy during the video conferencing sessions. As P7 explained,

“*Virtual brainstorming sessions are useful, but they rarely achieve the same level of spontaneity and energy as face-to-face discussions.”*

Besides, the study inferred that using digital technology successfully in collaborative instructional design necessitates important coordination as well as communication. Institute well-described roles and duties in faraway teams, and regular check-ins guarantee the progress of all members toward the desired goals. The results of a case study (Case Study 2, 2023) highlight that using project management tools is vital for ensuring alignment among team members and the timely accomplishment of tasks.

Furthermore, this study highlighted that having much reliance on such kind of framed technologies could result in communication fatigue due to which members of the team feel overwhelmed by the unceasing message flow and updates. In view of P8,

“*A continual stream or flow of updates, notifications and messages can engulf users, minimizing productivity rather than refining it.”*

Findings of the current study demonstrate that digital technologies have the capacity to underpin collaboration. Moreover, these technologies can encourage educators or learners to be creative in solving problems. Nonetheless, their remarkable achievement rests on transparent communication, effective collaboration, and the continued involvement of team members throughout the collaborative processes.

### 3.4 Challenges of integrating emerging technologies

Although emerging technologies have become useful tools in communication perspectives, their presence reflects some challenges as well. The integration of such technologies as artificial intelligence (AI), virtual reality, and augmented reality into the instructional design process facilitates outstanding opportunities in improving communication; however, at the same time, this incorporation also highlights new challenges. Augmented reality and virtual reality, although, can provide immersive learning experiences that assist educators or learners in visualizing concepts of instructional design; their proper usage relies on sufficient technical knowledge and expertise. One of the participants, P9, reported,

“*While virtual reality and augmented reality offer engaging learning opportunities, a great number of users have to face challenges in utilizing these technologies successfully because of inadequate technical awareness, training, and expertise.”*

Furthermore, AI supports the analysis of user feedback and the identification of patterns in instructional design, facilitating invaluable insights. Somehow, it also raised challenges regarding too much dependence on algorithm-centered decision-making. As P1 argued,

“*Artificial intelligence has the potential to support decision-making, but having dependence on it too greatly may minimize creativity and human judgment in the design process.”*

Likewise, according to P10, who had the same idea,

“*Algorithmic recommendations can facilitate significant support; however, they should complement professional-level expertise instead of replacing professional judgement.”*

The thematic analysis manifested that although developing technologies can enhance or improve communication, they also introduce new challenges that need careful management. A number of participants highlighted that the prominent learning loop linked with virtual as well as augmented reality might discourage their practical application, especially for customers and educators who are not familiar with such technologies. P11 reported his experience,

“*The users who have no familiarity with virtual reality (VR) and augmented reality (AR) may find it difficult to adopt these technologies due to a steep learning curve.”*

Equally, P3 suggested that insufficient experience with new technologies might restrict their wide-ranging usage in educational environments.

In addition, the usage of AI in instructional design and educational communication raises ethical issues regarding data privacy along with the potential for bias in algorithmic decision-making processes. From this perspective, participants indicated that artificial intelligence could serve as an influential tool for personalizing communication in different ways. Somehow, participants also stressed the significance of ensuring that the data used to train these algorithms do not show bias but are representative. One of the participants, P6, described,

“*Artificial intelligence makes it possible to adapt communication to individual needs; however, the transparency, fairness, and quality of its outcomes completely rest on the data used to train it.”*

Respectively, P1 reported his observation:

“*When training data comprises bias, AI-created decisions can probably depict those biases if no effective protections exist in their place.”*

Taken as a whole, the derived findings demonstrate that evolving technologies have the capacity to enforce communication and stakeholder participation in instructional design. Somehow, their successful usage rests on comprehensive professional judgement, technical skills, and careful attention to ethical considerations.

### 3.5 The role of visual communication in instructional design

In instructional management, the role of visual communication is significant. It facilitates designers in interpreting complex concepts in a clearer and more effective way. Visual communication plays a key role in the instructional design process by assisting instructional designers in proficiently interpreting complex concepts in a clear and effective way. Similarly, analysis of the collected data manifested that visual aids, including diagrams, sketches, and infographics, highly enhance engagement and understanding among educators. A case study manifested how an instructional designer uses a series of sketches to clarify the structure of a redesigned instructional design course to learners. As a result, learners were able to visualize the final instructional design, facilitating clearer and more meaningful feedback (Case Study 3, 2023).

Findings of the present study indicate that effective visual communication plays a vital role in improving the clarity and quality of graphics. Findings also highlight that it enhances the capability of the instructional designers to understand and respond to feedback. The interview-based data facilitated substantial support for this finding. Accordingly, P1 interpreted,

“*Visual representations (diagrams, sketches) assist in simplifying complex ideas and making communication more effective for both designers as well as learners.”*

Likewise, P4 observed,

“*Diagrams and sketches mostly communicate concepts in a clearer way as compared to lengthy written interpretations.”*

In addition to this, this study also suggested the importance of visual communication in overcoming language obstacles while promoting collaboration within diverse cultures. A great number of participants documented that visual aids are particularly valuable in international-level projects, where language differences can make communication more challenging. According to the observation of P13,

“*Visuals offer a common language that assists team members in communication, though they belong to diverse linguistic contexts.”*

Equally, P15 reported,

“*Images and diagrams minimize misunderstandings and support collaboration in multicultural teams.”*

Effective visual communication enables instructional designers to communicate their ideas in a way through which educators with diverse linguistic backgrounds can easily perceive and adopt them. Somehow, the analysis manifested that cultural differences could affect explanations related to images. Therefore, the findings suggest that instructional designers must be able to know about such delicacies while communicating with students from other countries. Accordingly, P1 remarked that

“*the visual image might be explained differently within the cultures, suggesting the need for instructional designers to think about cultural sensitivities while choosing visual elements.”*

As a whole, the results of this study underline that visual communication has a prominent role to play in improving insight, assisting the feedback process, and boosting collaboration across linguistic and cultural contexts. Somehow, its effectiveness depends on the clarity of visual materials and their suitability for different cultural contexts. In general, the findings indicate that visual communication works as a powerful mechanism aiming to enhance comprehension, support feedback processes, and offer collaboration within linguistic and cultural boundaries. However, the usefulness of visual communication relies on the clarity of visual materials as well as the cultural suitability of their design and presentation.

### 3.6 The impact of digital communication on user experience

Findings of the present study indicate that digital communication technologies exert a substantial impact on users‘ experiences by shaping how they participate in instructional design making and consider the instructional design processes. A case study, for instance, reflected the way through which an instructional designer utilizes an interactive prototype series to collect users' feedback on a new application. It facilitated the instructional designer to make iterative improvements depending on real user data (Case Study 4, 2023). The incorporation of digital technology led to a more refined instructional design while encouraging user interaction and supporting collaboration.

According to the participants, digital communication technologies make it easier for instructional designers and users to stay connected with each other throughout the process of designing, permitting feedback to be integrated more efficiently. As P14 explained,

“*Interactive digital tools permit users to offer feedback throughout the development process, making the final design more responsive to their needs.”*

Participants also observed that continuing communication assisted in refining instructional design solutions and encouraged greater user participation.

Somehow, the analysis highlighted several challenges associated with obtaining user feedback through digital technologies. A great number of participants judged that although evaluation of online surveys and remote usability offers convenience, they mostly lack the depth and attention to detail of face-to-face interactions. The management of online surveys and remote usability assessments may be appealing, but they mostly lack the meticulousness and carefulness of face-to-face interactions. As P14 explained,

“*Online surveys have the tendency to provide useful information, but they mostly fail in capturing the emotions and subtle reactions of the users.”*

One of the instructional designers, for instance, reported that although online surveys can offer enough data, they mostly fail to capture the emotional responses and subtle behaviors of the users as effectively as face-to-face observations. According to P14,

“*Face-to-face observations facilitate understanding of user behavior, but it becomes very difficult to obtain the same understanding through digital feedback mechanisms.”*

This result highlights that digital technologies, although they reflect many advantages, were perceived by participants as being more effective when aligned with traditional methods to ensure a systematic understanding of the learners' experiences. Taken together, the findings indicate that digital communication technologies may have the ability to increase interactivity and facilitate user participation; somehow, a balanced approach that incorporates traditional and digital communication methods is needed in order to achieve an inclusive understanding of users' needs and their experiences.

### 3.7 Ethical considerations in instructional design communication

The study's findings revealed that ethical considerations, especially in developing technologies, are essential in educational and instructional design communication. Usage of Artificial Intelligence (AI) in educational and design communication, for example, raises concerns about data privacy and the potential for algorithmic bias. Many participants showed their concerns about the ethical implications while using AI in order to personalize communication, specifically regarding the ways through which users' data are collected and used. In this regard, P1 claimed,

“*AI has the capability to personalize communication in an effective way, but users need to be aware of the way their data are collected, safely stored, and utilized in research.”*

P2 expressed his concern,

“*Privacy together with transparency should never be compromised while pursuing personalisation.”*

Similarly, one more participant observed that although AI can encourage the learner's experience by providing personalized direction, it is necessary to ensure that users are fully made aware of their data, their data usage, and the option to opt out. Another participant, P6, explained,

“*It is always right of the users to have awareness of how their provided information is being used, and the option to withdraw from data-driven systems if they prefer.”*

While proceeding further, findings also underscored the essentiality of inclusivity and accessibility in instructional design and educational communication. Some participants stressed the need for instructional designers to contemplate all users' different preferences and needs, as well as those having disabilities. According to P3,

“*Accessibility should be considered from the beginning of the design process rather than being added as an afterthought.”*

Likewise, P1 observed that

“*Inclusive communication requires designing for diverse users with different abilities, backgrounds, and learning needs.”*

For instance, one case study revealed how an instructional designer merged features of accessibility into a website instructional design, like alt text for images and keyboard navigation, to make sure that the site was available to use by individuals with visual and motor disorders/disability (Case Study 5, 2023).

Somehow, the analysis also disclosed that accessibility is often reconsidered in the instructional design process instead of being integrated from the start. This finding suggests that greater attention to the ethical implications of communication strategies may support more inclusive and accessible instructional designs. As P3 further remarked,

“*Ethical design is not only about technology; it is also about ensuring that no user is excluded from accessing and benefiting from the learning experience.”*

Overall, the findings suggest that ethical instructional design communication requires a balance between technological innovation, data privacy, transparency, accessibility, and inclusivity. Addressing such considerations is vital to ensure that developing technologies encourage equitable and responsible communication in educational setups.

## 4 Discussion

The results obtained from the thematic analysis of the current study accentuate the grave role of effective and influential communication in the instructional design process, chiefly from the perspective of digital tools as well as the act of storytelling. Unlike Duin and Pedersen (2023), who keep focusing on the performance and capability of AI-driven communication, this study highlights the trade-offs that exist between automation, mechanization, and human-focused dialogues. In this way, our findings after the thematic analysis recommend that instructional design teamwork is unable to rely merely on AI tools with unstructured or unorganized communication strategies, which can make creativity, spontaneity, and emotional engagement safe at all. The equilibrium between automation and discussions focused on humans affects how learning is transferred (Covello, 2025; Duin and Pedersen, 2023). Learners should actively engage with the material, rather than passively absorbing AI-generated content (Biju et al., 2024; Mayer, 2005). Studies show that thoughtfully integrating digital stimuli fosters more profound learning transfer (Rohles et al., 2022). The amalgamation of storytelling with instructional design, communication, and educational communication was discovered to be specifically effective in developing and improving stakeholder engagement and making a deeper understanding of instructional design concepts easier. Even so, the analysis of the present study also indicated that there remains an urge for instructional designers to improve well-built and well-structured communication skills to grasp and handle these techniques and tools efficiently (Vrontis et al., 2023).

Moreover, the findings have similarities and align with the revelations highlighted by Duin and Pedersen (2023), who proclaim that artificial intelligence mostly improves analysis based on instructional design feedback. The researchers of the present study also highlight a remarkable limitation: the deficiency of human awareness regarding AI-driven communication; this lack of awareness can create a hindrance to creative spontaneity within the group of instructional design teams and educational teams. If their research-based work highlights the technical benefits of artificial intelligence, our research has underscored a remarkable limitation, respectively. In addition, though Rajaratnam et al. (2022) consider and perceive augmented reality and virtual reality as leading instruments for engagement, our data discloses an ease of access to the challenges that hamper their extensive use. Accordingly, the researchers offer a hybrid communication pattern that assimilates digital technology with narrative tactics in order to heighten the lucidity of remote collaboration among the community people (Pohn et al., 2025).

The findings of the study depict several practical implications for instructional design practitioners. Instructional designers should be acquainted with the limitations of digital tools and be able to take measures to ensure communication is clear and effective. For instance, managing clear communication protocols and expectations can assist in mitigating the possibility of miscommunication in isolated teams. Furthermore, the integration of storytelling with communication-based strategies can increase stakeholder engagement and lead to better instructional design results (Kim and Li, 2021; Kenny and Castilla-Rho, 2022; Ajabshir, 2024). Educators should employ scaffolding techniques, such as reflective prompts and timely feedback, to enhance learners' self-regulation in virtual design activities (Gopez and Gopez, 2024; Zimmerman, 2002). Likewise, instructional designers must also be conscious of the challenges connected with emerging technologies and make certain that these tools are used in ways that develop, rather than hinder, the communication process.

The present study equally points out the importance and value of considering principled insinuations in instructional design communication and educational communication. The designers should be animated and active in making certain that the communication strategies they manage are all-inclusive and controllable to all users. Moreover, they should show reverence for the self-sufficiency and confidentiality of users while using evolving technologies such as AI and other digital communication sources.

In the same way, the findings of the current research have remarkable suggestions for the advancement and improvement of the futuristic theoretical frameworks closely related to effective design communication and educational communication. The thematic analysis of the study also revealed the requirement for a further all-encompassing understanding of the utilization of digital tools and storytelling techniques. Moreover, the findings will assist in understanding how these techniques impact creativity as well as collaboration in distance teamwork. Existing reflective practice frameworks may not fully capture the interactive and fast-changing nature of digital communication tools, so updated models are needed to integrate both their benefits and challenges, along with the ethical considerations involved (see Sandars et al., 2024; Schön, 2017; Oates and Mynott, 2025). This study proposes that updated models are required to integrate the challenges and benefits of digital tools, as well as the ethical considerations associated with their usage. Theoretical advancements may combine self-regulated learning frameworks (Gopez and Gopez, 2024; Zimmerman, 2002) with social presence theory to create a more comprehensive model for digital instructional design communication (Barbosa, 2024; Garrison, 2007).

Based on these results, the present study suggests the Hybrid Storytelling–Digital Communication (HSDC) Model. The purpose of employing this model is to facilitate instructional design teams working within geographically dispersed environments. This proposed model in the current study combines structured storytelling techniques with digital communication technologies in order to address common challenges and limitations associated with virtual collaboration. The suggested model has been specifically designed in order to lessen the loss of non-verbal cues, encourage more natural communication, and develop meaning-making dialogue that is not easily achievable through online systems. Furthermore, in future studies, this employed strategy has the potential to be empirically assessed, aiming to examine its appropriateness and implementation within several instructional design situations. Since the study is based on thematic analysis, it facilitates initial assistance for the recommended HSDC model by categorizing frequent patterns linked with digital communication. Additionally, it also develops technologies, storytelling techniques, collaboration, and users' experiences along with communication based on ethics.

The present study also underscores the need for developing theories or theoretical frameworks that reflect the evolving and diverse nature of communication in instructional design and education. Accordingly, as digital technologies continue to evolve, introducing new working modes, future research should investigate how digital tools frame not just instructional design processes but also the larger social as well as cultural contexts in which they are used.

Furthermore, the results of the current study show alignment with the recent research conducted on AI-mediated communication and digitalization-based learning settings. These findings correspond with the observations of Duin and Pedersen (2023), proposing that Artificial Intelligence (AI) has the strength to bring improvement in communication processes, assisting feedback assessment. In contrast to Duin and Pedersen's (2023) emphasis on technological proficiency, results of this study underscore the continuing importance of human-centered communication, emotional involvement, and creative spontaneity within the work of instructional design teams. Equally, being consistent with Rajaratnam et al. (2022), these findings highlight that technologies based on virtual and augmented reality can improve a user's engagement. Nonetheless, participants also indicated challenges related to technical skills, accessibility and implementation, highlighting that these technologies need careful planning and proper support for effective adoption. The findings also align with Ajabshir (2024), who reported that storytelling can enhance learner engagement and retention. Similarly, the present study found that storytelling improved stakeholder understanding, emotional connection, and communication effectiveness. Consistent with Zhang and Zhang (2024), participants highlighted the challenges of remote collaboration and reduced social presence in digital environments. Overall, while recent studies have largely emphasized the benefits of digital technologies, the present study extends existing knowledge by proposing the HSDC model, which integrates AI-assisted storytelling with human-centered communication practices to support collaboration and engagement in instructional design contexts.

### 4.1 Limitations

As far as this study is concerned, it provides valuable perceptions related to the role of communication in instructional design; however, several limitations should be acknowledged. The use of a qualitative research design, while well suited to the exploration of complex phenomena, means that these findings may not be generalized to all instructional design contexts (Lincoln and Guba, 1985; Bingham, 2023). Besides, having dependence on self-reported data obtained from interviews and case studies may reflect response and recall bias, since participants may not continually and accurately recall or report their personal experiences (Podsakoff et al., 2003; Zhang and Zhang, 2024).

There is another limitation, which indicates that the pilot implementation of the HSDC model consisted of just six participants. The pilot application was intended to explore the initial feasibility and practical implementation of the proposed model rather than to provide statistical validation or formal empirical testing. Future studies involving larger samples are needed to validate the model across different educational contexts (Sandars et al., 2024; Trevisan et al., 2024; Oates and Mynott, 2025).

Correspondingly, the current study focuses initially on the individual experiences made by AI developers, educators, instructional designers, learners, as well as stakeholders working within digital and technology-centered setups. Since the current study focused on sectors that actively utilize digital communication tools and developing technologies, its analysis may not be fully applicable to instructional design situations like industrial design, architecture, or fashion (Hofstede, 2001; Wei et al., 2025). Future research should therefore explore communication strategies across a broader range of instructional design disciplines to provide a more comprehensive understanding of communication practices in instructional design.

Moreover, cultural differences and educational psychology frameworks may influence how communication strategies, storytelling approaches, and digital technologies are interpreted and applied across different contexts (Hofstede, 2001). Recent studies continue to emphasize the importance of learners' cultural backgrounds in shaping technology acceptance, engagement, and communication preferences (Zhang and Zhang, 2024).

### 4.2 Future research directions

The study has identified many areas for future research. Firstly, there remains a need to explore how developing technologies such as AI can be merged with instructional design, communication, and educational communication strategies. Moreover, there is a need to know the ways that assist instead of replacing human judgment and creativity. Additionally, further research is required on the best practices to manage remote teamwork in instructional design, especially in terms of upholding creativity as well as cohesion in dispersed teams. Ultimately, future studies should contemplate developing up-to-date theoretical frameworks that better support the interactive and dynamic nature of modern communication tools (Zhou et al., 2017; Kang, 2022) in instructional design.

Another significant area for future research is the investigation of communication strategies in a larger range of instructional design disciplines. While the current study has focused initially on the digital and tech industries, it is necessary to understand how communication strategies and challenges differ across the fields, i.e., fashion, architecture, and industrial design. However, the current study has the capacity to offer valuable insights into how several industries reflect their tendency to approach communication in the design process, and how these strategies can be modified to meet the specific requirements of each field.

### 4.3 Implications for practice

For instructional design practitioners, the present study highlights the importance of effective communication in achieving successful instructional design outcomes. For instructional design practitioners, the current study emphasizes the significance of effective communication in achieving successful instructional design outcomes. It is also necessary for instructional designers to be aware of the limitations of digital tools. They should take measured steps to ensure that communication is clear and fully concentrated. Furthermore, incorporation of storytelling with communication strategies can encourage stakeholders' participation; it can also lead to better instructional design results. In this perspective, changing technologies keep bringing evolution in their advancement; therefore, it becomes necessary for instructional designers to keep themselves updated, creative, adaptable, and innovative in this field. Moreover, they will require adopting the current communication approaches that tend to boost creativity, alliance, and participation of the stakeholders.

Findings of the study also highlight the necessity of focusing on ethical implications in both types of communication, design communication and educational communication. It is the job of an instructional designer to be active in ensuring that their communication approaches are all-encompassing, wide-ranging, and easy to access for all users, and that they take care of the privacy and sovereignty of users while using emergent technologies like Artificial Intelligence (AI).

## 5 Conclusion

This study aimed to examine how AI-assisted storytelling and digital communication technologies influence instructional design communication, collaboration, stakeholder engagement, and learner interaction. The present study identified the fundamental and significant role of communication in shaping impressive design experiences, with a definite focus on the incorporation of digital tools with the art of storytelling. The analysis also indicates both the benefits as well as challenges associated with the application of digital tools in the instructional design process and the significance of storytelling art in improving stakeholder engagement or participation. In this study, the theme-based analysis reflected several main themes. It also revealed the impact of digital tools on the communication held by instructional designers and learners. Moreover, the findings demonstrated the challenges related to collaboration and creativity in the digital era, the role of storytelling in instructional design, and the ethical challenges concerning technologies of the future. Drawing on the principles of educational psychology together with cognitive load, self-regulation, and motivation can assist instructional designers in tailoring digital storytelling interventions to bring improvement in learning outcomes.

Analysis of the present study explored the ways through which storytelling communication tools mediated by AI facilitate collaboration while responding to challenges in isolated instructional design and ethical challenges. Similarly, these tools address concerns in collaboration and in AI-assisted educational communication. Furthermore, the findings demonstrated how digital communication technologies, storytelling approach, and AI exert an impact on instructional design involvement. These findings further identify challenges concerning accessibility, ethical usage, and the absence of non-verbal cues while indicating the importance of digital tools and storytelling approach in strengthening communication, creativity, cooperation, and stakeholder participation.

In the analysis, it has been observed that the increasing incorporation of communication and instructional design underscores the requirement for responsiveness as well as adaptability in the present-day instructional design practices. Therefore, due to continuing advancement in digital tools and storytelling strategies, instructional designers are required to remain more alert. Accordingly, they should be ready to adopt and utilize flexible and innovative communication approaches that support creativity, cooperation (teamwork), and purposeful participation of the stakeholders. Due to these efforts, educational communication and instructional design have the capacity to manage collaboration, innovation, and create useful changes. Besides, when instructional design keeps adopting developing technologies, it will become necessary for practitioners to restructure their communication approaches and skills so that they are able to successfully navigate the complexities found in the current instructional design settings.

However, in future research, the HSDC model has a prominent position. Therefore, the researchers should empirically assess and validate this model; they should also investigate the impact it exerts on instructional design creativity, usefulness, collaboration, and stakeholder participation throughout different cultural contexts. By bringing advancements in robust communication skills, having consciousness of ethical challenges or considerations, and adopting new storytelling approaches or techniques, instructional designers have the capacity to provide more effective and purposeful instructional design experiences. Collectively, design communication and educational communication, especially through these efforts, will be able to continue to change while shaping the future of instructional design.

## Data Availability

The original contributions presented in the study are included in the article/supplementary material, further inquiries can be directed to the corresponding author.
