# Supplementary material for: Instructional design communication through AI-assisted storytelling: a hybrid model grounded in educational psychology
Source: Front Psychol. 2026 Sep 3;17:1785568. doi: 10.3389/fpsyg.2026.1785568 (PMC13582278; doi:10.3389/fpsyg.2026.1785568)
Supplement: Supplementary file 1 [file Supplementary_File_1.docx]

**Appendix ASupplementary**

**Semi-structured Interview Protocol**

The interview guide/themes used to structure the semi-structured interviews

**Section A. Background Information**

1. Please describe your role in instructional design, education, AI development, or related activities.
2. How long have you been involved in instructional design or educational communication activities?

**Section B. Digital Communication in Instructional Design**

3. How do digital communication tools influence collaboration in instructional design projects?
4. What advantages and challenges have you experienced when communicating through digital platforms?

**Section C. Storytelling and Engagement**

5. How does storytelling influence communication with learners, educators, or other stakeholders?
6. Can you describe a situation where storytelling improved understanding or engagement?

**Section D. Collaboration and Creativity**

7. How do digital communication tools affect creativity and teamwork during instructional design activities?
8. What communication practices support effective remote collaboration?

**Section E. Emerging Technologies**

9. What role do AI, virtual reality, or augmented reality play in instructional design communication?
10. What challenges do you encounter when using these technologies?

**Section F. Visual Communication**

11. How do visual elements such as diagrams, sketches, infographics, or prototypes support communication?
12. Have visual communication tools helped overcome language or cultural barriers? If so, how?

**Section G. User Experience and Feedback**

13. How do digital technologies influence the collection and use of learner feedback?
14. What differences do you observe between digital and face-to-face feedback processes?

**Section H. Ethical Considerations**

15. What ethical concerns arise when using AI and digital technologies in instructional design communication?
16. How can accessibility, inclusivity, and privacy be improved in instructional design communication?

**Closing Question**

17. Is there anything else you would like to add regarding communication, storytelling, AI, or instructional design practices?
